# Pharmacometric Analysis of Cafedrine/Theodrenaline Versus Ephedrine on Maternal Hemodynamics and Neonatal Acidosis During Cesarean Section

**DOI:** 10.3390/pharmaceutics18030296

**Published:** 2026-02-27

**Authors:** Christiane Dings, Thorsten Lehr, Peter Kranke, Benjamin Vojnar, Christine Gaik, Tilo Koch, Leopold Eberhart, Susanne Huljic-Lankinen, Melanie Murst, Sascha Kreuer

**Affiliations:** 1 Saarmetrics GmbH, 66123 Saarbrücken, Germany; thorsten.lehr@uni-saarland.de (T.L.); sascha.kreuer@uks.eu (S.K.); 2 Clinical Pharmacy, Department of Pharmaceutical Science, Saarland University, 66123 Saarbrücken, Germany; 3 Department of Anaesthesiology, Intensive Care, Emergency and Pain Medicine, University Hospital Würzburg, 97080 Würzburg, Germany; kranke_p@ukw.de; 4 Department of Anesthesiology and Intensive Care Medicine, University Hospital Giessen and Marburg, Campus Marburg, Philipps University of Marburg, 35043 Marburg, Germany; 5 Department of Anesthesiology and Intensive Care, Philipps University Marburg, 35043 Marburg, Germanyeberhart@staff.uni-marburg.de (L.E.); 6 Ratiopharm GmbH, 89079 Ulm, Germany; susanne.huljic-lankinen@ratiopharm.de (S.H.-L.); melanie.murst01@teva.de (M.M.); 7 Department of Anesthesiology, Intensive Care and Pain Therapy, Saarland University Medical Center, 66421 Homburg, Germany

**Keywords:** anesthesiology, cafedrine, cesarean section, ephedrine, hemodynamics, hypotension, obstetrics, theodrenaline

## Abstract

**Background/Objectives**: Ephedrine and cafedrine/theodrenaline (C/T) are established treatments for spinal anesthesia-induced hypotension during cesarean section. Both aim to stabilize maternal blood pressure and enhance neonatal oxygenation. We compared their effects on maternal hemodynamics and neonatal acid-base status using population kinetic/pharmacodynamic (K/PD) modeling and multiple regression analysis. **Methods**: The multicenter, prospective, open-label, two-armed, non-interventional HYPOTENS study included 243 parturients undergoing spinal anesthesia for elective cesarean section in Germany. Hypotension was treated with intravenous boluses of either C/T (10–200 mg, 55.6%) or ephedrine (5–40 mg, 44.4%), with dosing determined by the attending anesthesiologist. Maternal mean arterial pressure (MAP), systolic blood pressure (SBP), and heart rate (HR) were recorded for 30 min after treatment. Neonatal acidosis biomarkers included umbilical arterial pH, base excess (BE), and lactate. **Results**: A population K/PD model captured an initial increase followed by a plateau in MAP, SBP and HR after treatment. Maximum HR (MAX_HR_) was 15% higher after ephedrine than after C/T (*p* < 0.001). BMI and spinal block height significantly influenced maternal hemodynamics (both *p* < 0.001). Neonatal biomarkers were associated with the duration of maternal MAP below pre-surgery levels, gestational age, spinal block height, antihypotensive treatment, bupivacaine dose, and MAX_HR_ (all *p* < 0.05). **Conclusions**: Ephedrine was associated with higher maternal MAX_HR_. Notably, higher maternal MAX_HR_ was correlated with lower neonatal BE, suggesting that lower maternal peak HR may benefit. These findings may support the use of substances that are largely inert with respect to maternal HR.

## 1. Introduction

Spinal anesthesia-induced hypotension (SAH) during cesarean section poses risks to both maternal and fetal well-being [1,2]. In the mother, SAH may cause nausea, dizziness and vomiting; in the fetus, reduced uteroplacental perfusion can contribute to acidosis, reflected by reduced base excess (BE) and elevated lactate [2,3].

Both the 20:1 combination of cafedrine and theodrenaline (C/T) and ephedrine are vasoactive agents used to treat SAH, commonly administered as an intravenous (IV) bolus [4]. Cafedrine and theodrenaline act synergistically to increase cardiac output and mean arterial pressure via β1-adrenoceptor stimulation and phosphodiesterase inhibition, while generally producing only a moderate chronotropic effect [5]. After IV administration, onset occurs within approximately one minute and effects typically persists for 10–20 min [5]. Ephedrine is a sympathomimetic agent via α- and β-adrenergic activity, leading to increased cardiac output and heart rate (HR) [5].

During cesarean section, antihypotensive treatment aims to restore maternal blood pressure and to support fetal oxygenation [4]. However, direct comparisons of the hemodynamic effects of C/T and ephedrine remain limited [5,6,7]. Many existing studies report outcomes at discrete time points following single-dose administration, which does not fully reflect the dynamic, real-time dosing and re-dosing decisions made in routine clinical practice [6,8].

Mathematical modeling can help address these limitations. Non-linear mixed-effects (NLME) models enable characterizations of drug effects over time while accounting for different dose amounts, repeated dosing, inter-individual variability (IIV), and the influence of patient-specific factors [9,10].

In this study, we applied kinetic/pharmacodynamic (K/PD) NLME modeling to characterize the effects of C/T and ephedrine on mean arterial pressure (MAP), systolic blood pressure (SBP), and HR after single or repeated dosing during cesarean section. We also investigated the influence of intrinsic and extrinsic factors, including age, pregnancy duration, and spinal block height. Additionally, we performed multiple regression analyses to assess associations between maternal hemodynamics and neonatal outcomes umbilical arterial (UA) pH, BE, and lactate.

## 2. Materials and Methods

### 2.1. Study Design

The HYPOTENS study was a prospective, open-label, two-armed, non-interventional study conducted at multiple centers in Germany. Between July 2016 and February 2018, patients were recruited to evaluate the routine clinical use of C/T (cafedrine hydrochloride 200 mg/theodrenaline hydrochloride 10 mg per 2 mL solution for injection, ratiopharm GmbH, Ulm, Germany) and ephedrine (ephedrine hydrochloride 10 mg per 1 mL solution for injection, Sintetica GmbH, Münster, Germany).

For the present analysis, data were used from parturients receiving C/T or ephedrine for the treatment of SAH during elective cesarean section (n = 283 per-protocol patients). Hypotension was defined as SBP < 100 mmHg or <90% of the pre-operative baseline SBP. Patients who received prophylactic antihypotensive treatment were excluded.

The timing, dose, and any repeated administration of the study drugs were determined at the discretion of the attending anesthesiologist. Maternal hemodynamic parameters, including MAP, SBP, and HR, were recorded immediately before the first antihypotensive bolus (baseline at hypotension diagnosis) and t 1, 2, 3, 4, 5, 6, 7, 8, 9, 10, 12, 15, 20, 25, and 30 min after the first dose, irrespective of the time of delivery. Neonatal biochemical outcomes were assessed using UA pH, BE, and lactate as surrogate markers of fetal acid-base status. No pharmacokinetic (PK) samples were obtained during this study.

The primary study endpoints were (i) the area under the curve (AUC) of the deviation between observed SBP and the individual target SBP and (ii) the incidence of newly occurring tachycardia (HR ≥ 100 bpm). Detailed information on the study procedures, recruitment, and primary results have been previously published [4,6].

### 2.2. Data Analysis

Dataset generation, statistical analyses, and graphical visualization were performed using R version 4.3.0 (The R Foundation for Statistical Computing, Vienna, Austria). Maternal hemodynamic modeling was performed using NLME methods in NONMEM version 7.4.3 (ICON Development Solutions, Ellicott City, MD, USA). Model selection was guided by the objective function value (−2 log likelihood), precision of parameter estimates (reported as relative standard errors, RSE), and visual inspection of the goodness-of-fit (GOF) plots and conditional weighted residuals vs. time [11,12]. Parameters were estimated using first-order conditional estimation with interaction.

A K/PD model was developed to describe the hemodynamic parameters SBP, MAP, and HR following administration of C/T and ephedrine.

In the absence of PK data and published PK models for cafedrine, theodrenaline and ephedrine, we applied a K/PD approach in which drug exposure is represented by a hypothetical kinetic compartment [13]. A one-compartment structure with first-order elimination was selected as the most identifiable model capable of describing the characteristics of each drug’s action from the pharmacodynamic (PD) data alone. To ensure identifiability, the apparent volume of distribution (V) was fixed to 1 L. In this K/PD framework, V serves only as a scaling parameter. Hence, V does not imply a physiological distribution volume. Kinetic parameters should therefore be interpreted as functional descriptors of response dynamics rather than systemic pharmacokinetics.

To link the kinetic and pharmacodynamic components, we evaluated direct-effect models and delayed-effect models implemented via transit compartments. We selected the simplest structure that captured the onset and smoothing of the response while maintaining acceptable parameter precision. We tested multiple configurations, including different numbers of delay compartments and transit rates for each hemodynamic parameter, as well as combined effect and tolerance models. Finally, to link the concentration of the delay compartments to the hemodynamic parameters, different effect models, such as linear, Emax, and hill models, were tested.

IIV was evaluated for all model parameters using exponential models. Additive, proportional, and combined additive and proportional error models were tested to account for unexplained residual variability.

We assessed the following covariate categories: (i) Maternal demographics (age, weight, height, BMI, ASA grade, hypertension diagnosis, gestational diabetes, and pregnancy duration); (ii) baseline and pre-surgery hemodynamics (SBP, diastolic blood pressure (DBP), MAP, and HR); and (iii) anesthesia- and delivery-related variables (bupivacaine dose, baricity, spinal block height, opioid treatment (yes/no, which opioid and dose), reason for cesarean section (primary/secondary), degree of left side positioning, and blood loss).

Covariate candidates were initially screened for correlation with the empirical Bayes estimates (EBEs) of the model parameters. Covariates showing significant associations (*p* < 0.05) were then introduced univariately into the NLME model through univariate forward inclusion (*p* < 0.05) and then subjected to backward elimination (*p* < 0.001). Continuous covariates were modeled using power functions centered at the population median, as specified by Equation (1):

(1) $$Effect={\left(COV/\bar{COV}\right)}^{\theta }$$

with the individual covariate value COV, the population median $\bar{COV,}$ and the estimated exponent θ. Categorical covariates were modeled as fractional changes relative to a reference category, as shown in Equation (2).

(2) Effect=1+COV×θ

with the individual covariate level COV and the estimated effect size θ.

The final covariate model was additionally evaluated for the influence of major intraoperative events (anesthesia, incision, and uterotomy). We also assessed whether treatment group influenced structural model parameters that included IIV.

To identify independent predictors of neonatal outcomes (UA pH, BE, and lactate), we conducted stepwise multiple linear regression analyses using the “stepWise” function from the mixlm R package (Version 1.3.0; Liland 2023) [14]. An F-test-based model-effect selection process was employed, using a forward selection (*p* = 0.1) and backward elimination (*p* = 0.05) procedure. Candidate predictors included: all variables considered in the maternal hemodynamic modeling; individual parameter estimates from the maternal hemodynamic model; the neonate’s weight and sex; and cesarean section-related parameters including (i) time from anesthesia, uterotomy and treatment to cord clamping, (ii) time from cord clamping to blood sampling, and (iii) the duration and AUC of MAP and SBP below pre-surgery levels, respectively. Newborns were considered untreated if cord clamping occurred before the parturient received C/T or ephedrine.

### 2.3. Simulations

The final models were used to simulate the effect of different C/T and ephedrine doses on maternal hemodynamics and newborn outcomes. Simulations were performed for a “typical” parturient defined by the median covariate values of the study population. For neonatal biomarker simulates, the duration of maternal MAP below the pre-surgery MAP (TIME_MAP_) was calculated from the simulated MAP-time profiles. If the MAP at the average time of cord clamping remained below the pre-surgery MAP, TIME_MAP_ was defined as the median time interval between drug administration and cord clamping.

Simulations were repeated 1000 times, incorporating IIV but excluding residual variability. Simulation results were summarized as medians and 10th and 90th percentiles.

## 3. Results

### 3.1. Dataset

For the present analysis, participants from the per protocol analysis set (n = 283) were excluded if they experienced blood loss exceeding 1000 mL during the operation (n = 9, 3.2%), were treated with hydroxyethyl starch, albumin, gelatine, fresh frozen plasma, or red blood cell concentrates (n = 19, 6.7%), underwent a change to general anesthesia (n = 1, 0.4%), and/or received local anesthetics other than bupivacaine (n = 15, 5.3%). These exclusions were made because such events were expected to substantially confound the hemodynamic profiles. In total, 40 patients (14.1%) were excluded, yielding a final dataset of 243 parturients, of whom 135 (55.6%) received C/T and 108 (44.4%) received ephedrine.

Parturients had a mean (SD) age of 33.4 ± 5.22 years, BMI of 31.6 ± 6.58 kg/m^2^, bodyweight of 87.6 ± 19.7 kg, and height of 166 ± 6.91 cm. The most common single doses were 50 mg for C/T (range 10–200 mg per dose) and 15 mg for ephedrine (range 5–40 mg per dose). Over the 30-min observation period, the median cumulative dose was 100 mg C/T (range 20–400 mg) or 30 mg ephedrine (range 10–120 mg). Newborns had a median weight of 3370 ± 547 g and gestational age of 262 ± 10.2 days. Demographic characteristics summarized by treatment group are presented in Table 1.

UA pH, BE, and lactate measurements were available for 228 (93.8%), 205 (84.4%), and 76 (31.3%) newborns, respectively. Newborns were classified as untreated if cord clamping occurred before administration of C/T or ephedrine to the mother, which applied to ten pH (4.4%), eight BE (3.9%) and one lactate (1.3%) measurement.

Maternal hemodynamics, newborn outcomes, and surgery-related parameters are summarized in Table 2. The term “pre-surgery” refers to the last values recorded before anesthesia, whereas “baseline” refers to values measured at the time of hypotension diagnosis.

### 3.2. Maternal Hemodynamics

**Figure 1:**
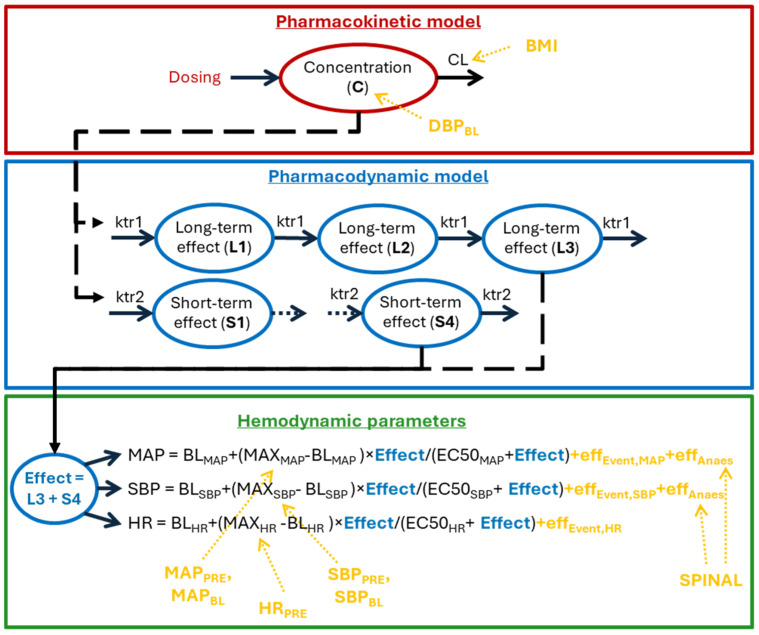
Schematic representation of the final model. The final model comprises a one-compartment kinetic model (red), a pharmacodynamic model (blue) representing the delay between treatment and effect, and the description of each hemodynamics parameter (green). Full arrows indicate mass transfers. Dashed arrows indicate influences. The impact of covariates is marked in yellow. BL = baseline; CL = clearance; eff_Anaes_ = anesthesia effect; eff_Event_ = effect of incision and uterotomy, HR = heart rate; ktr1 + ktr2 = transit rates; MAP = mean arterial pressure; MAX = Maximum impact; PRE = presurgery; SBP = systolic blood pressure.

A K/PD model was developed describing MAP, SBP, and HR responses to C/T and ephedrine (Figure 1). In the absence of PK data, a one-compartment kinetic component with first-order elimination was used. The estimated half-life for both drugs was 7.14 min and decreased with increasing maternal BMI. The apparent volume of distribution was fixed to 1 L and increased with lower baseline DBP. To capture onset and persistence of drug effects, four and three transit compartments were incorporated, with mean transit times of 2.27 and 28.0 min, respectively.

The hemodynamic effects were best described using the Emax model, driven by the concentrations in the final transit compartment. Linear models did not adequately capture saturation at higher exposure levels, whereas the Emax model provided a superior description of the exposure–response relationship with improved fit diagnostics and physiologically plausible behavior across the dose range. Population estimates for the maximum effects were 120 mmHg for maximum MAP (MAX_MAP_) and 169 mmHg for maximum SBP (MAX_SBP_). MAX_MAP_ and MAX_SBP_ increased with higher pre-surgery values and lower baseline values, respectively. The population estimate for maximum HR (MAX_HR_) was 77.9 bpm after C/T and 89.6 bpm after ephedrine, and increased with higher pre-surgery HR.

IIV was identified for clearance, the apparent volume of distribution, and the maximum effect parameters, with pre-surgery and baseline hemodynamic parameters among the most influential covariates. For C/T, EC50 values were 61.4 (MAP), 64 (SBP) and 5.6 (HR) mg/L, consistent with rapid HR effect saturation. EC50 values for ephedrine were 71.1% lower than those for C/T (17.7, 18.5 and 1.6 mg/L, respectively), indicating comparable blood pressure effects for 50 mg C/T and 15 mg ephedrine.

Following anesthesia, MAP and SBP dropped on average by 9.79 mmHg with larger decreases observed at lower thoracic block levels. At incision and uterotomy, MAP, SBP, and HR increased by 0.8 mmHg, 2.39 mmHg, and 2.75 bpm, respectively.

**Figure 2:**
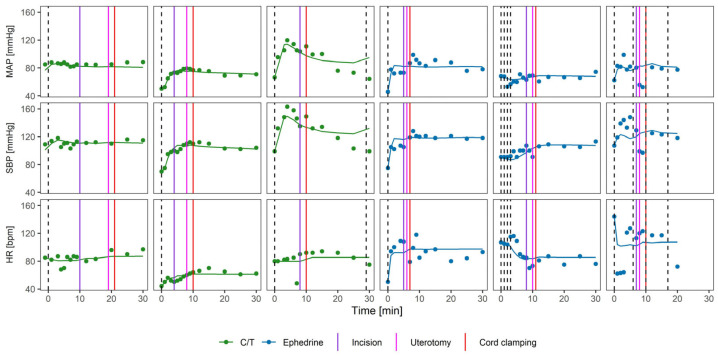
Exemplary individual profiles. Points represent observations and lines represent individual model predictions. Dashed vertical lines indicate dosing events. Full vertical lines indicate other events.

**Figure A1:**
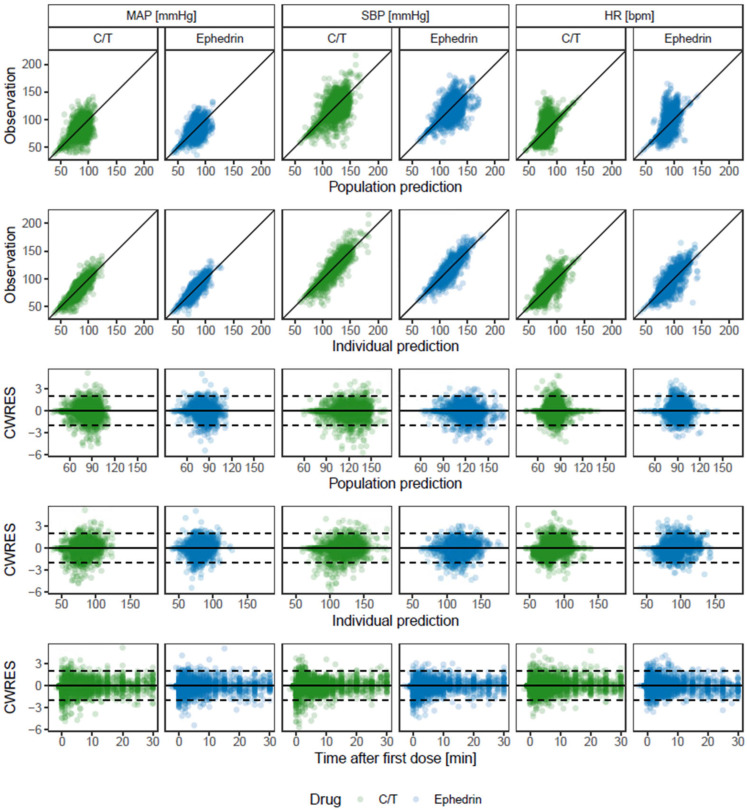
Goodness-of-fit plots for MAP, SBP and HR stratified by treatment. Full lines indicate lines of identity. Dashed lines indicate CWRES of −2 and 2. C/T, cafedrine/theodrenaline; CWRES, conditional weighted residuals; HR, heart rate; MAP, mean arterial pressure; SBP, systolic blood pressure.

Figure 2 shows hemodynamic profiles of six randomly selected parturients following administration of C/T or ephedrine. Model equations are provided in Appendix A, and parameter estimates including covariate significance are summarized in Appendix A.1 Table A1. GOF plots demonstrate an accurate model performance without evident trends or biases (Appendix A.1 Figure A1).

**Figure 3:**
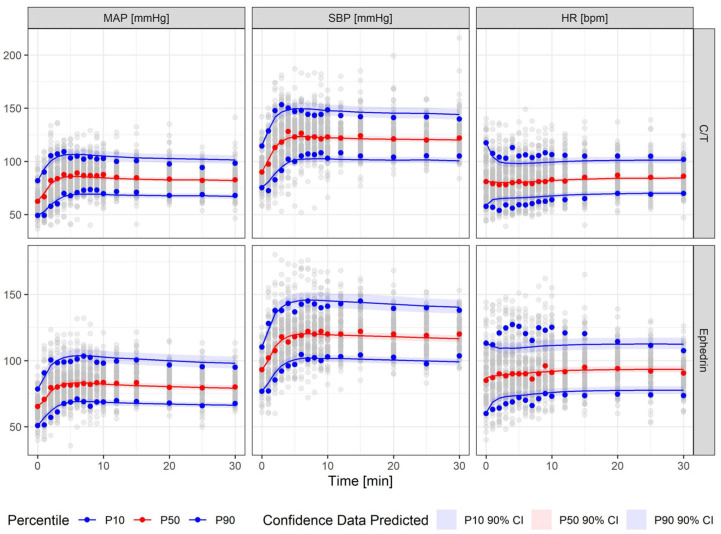
Visual predictive check. Grey points represent all observations. Colored points represent the 10th, 50th and 90th percentiles of observations, lines represent corresponding model predictions and bands represent the corresponding 90% confidence intervals.

**Figure A2:**
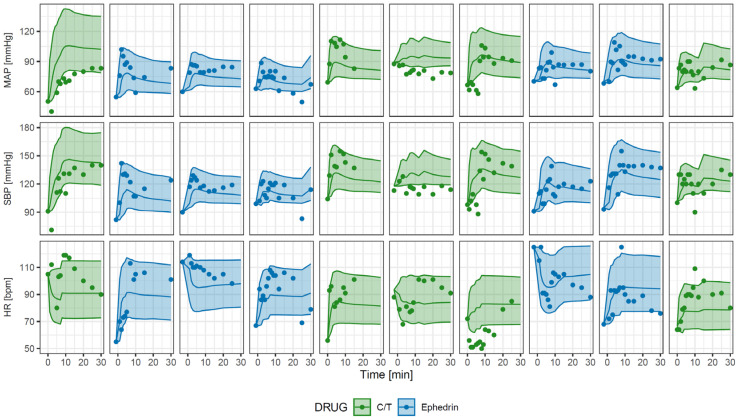
Observations and model simulations for 10 randomly selected study participants. Points represent observations, lines represent the median of 1000 simulations, bands indicate the 90% prediction interval for each patient.

For model validation, a visual predictive check (VPC) was performed, stratified by treatment arm. Each subject was simulated 1000 times. Simulation results are shown as 10th, 50th and 90th percentiles with 90% confidence intervals, and overlain with the corresponding observed percentiles. As shown in Figure 3, the model described the observed profiles well. However, for HR, variability in the 10th and 90th percentiles was high and could not be captured fully. Appendix A.1 Figure A2 presents observed data and model simulations for five randomly selected patients per treatment group.

### 3.3. Newborn Outcome Biomarkers

**Figure A3:**
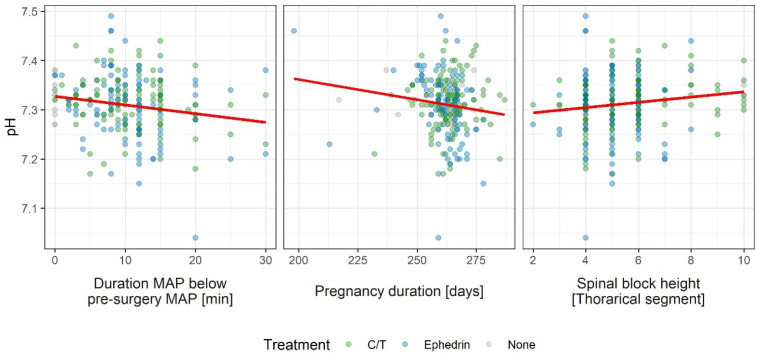
pH observations (points) and model predictions (lines) for univariate changes in each predictor. The respective other predictors were set to median levels.

**Figure A4:**
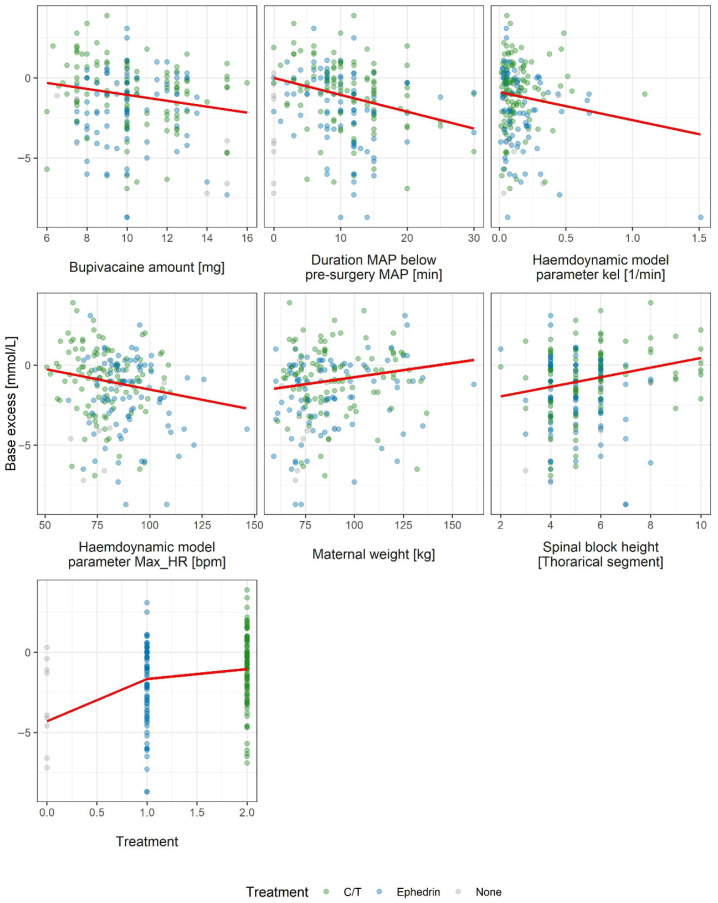
Base excess observations (points) and model predictions (lines) for univariate changes in each predictor. The respective other predictors were set to median levels. Treatment: 0 = none, 1 = ephedrine, 2 = C/T.

**Figure A5:**
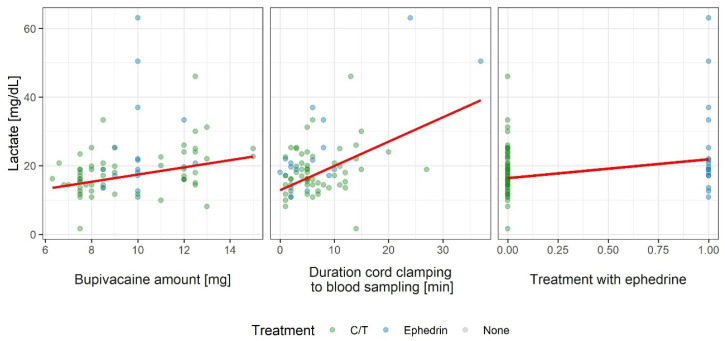
Lactate observations (points) and model predictions (lines) for univariate changes in each predictor. The respective other predictors were set to median levels. Treatment with ephedrine: 0 = no, 1 = yes.

Stepwise multiple linear regression identified predictors of newborn outcome biomarkers UA pH, BE, and lactate using individual maternal hemodynamic model parameter estimates and other covariates described in Appendix A. Regression estimates, RSEs, and significance levels are provided in Appendix A.2 Table A2, Table A3 and Table A4. Observed versus predicted relationships are shown in Appendix A.2 Figure A3, Figure A4 and Figure A5.

UA pH decreased by 0.00177 per minute, and maternal MAP remained below the pre-surgery MAP until cord clamping (*p* = 0.0613) and by 0.000822 per additional pregnancy day (*p* = 0.0298). UA pH also decreased by 0.000822 per additional day of gestation (*p* = 0.0298). Spinal block height (modeled as a continuous covariate) was associated with a decrease in pH of 0.00534 per additional spinal segment (*p* = 0.0309).

Compared with untreated newborns, BE was 2.64 mmol/L higher for newborns of mothers treated with ephedrine and 3.26 mmol/L higher for newborns of mothers treated with C/T (both *p* < 0.001). BE decreased by 0.105 mmol/L for each minute of MAP below pre-surgery MAP (*p* < 0.001), by 0.3 mmol/L for each increasing spinal segment (*p* = 0.00134), by 0.186 mmol/L per mg bupivacaine (*p* = 0.00536), by 0.0256 mmol/L per bpm of the hemodynamic model parameter MAX_HR_ (*p* = 0.0202), and by 0.0256 mmol/L per min/L increase in the elimination rate of C/T or ephedrine (*p* = 0.0368). BE increased by 0.0176 mmol/L per kg body weight of the mother (*p* = 0.0171).

Lactate was 5.46 mg/dL higher in newborns of mothers treated with ephedrine compared with those treated with C/T (*p* = 0.00883). Lactate increased by 0.708 mg/dL per minute between cord clamping and blood sampling (*p* < 0.001) and by 1.05 mg/dL per mg bupivacaine administered for spinal anesthesia (*p* = 0.00967).

### 3.4. Simulations

Simulations were performed for single doses of the most commonly administered doses (40, 50, and 100 mg C/T and 10, 15, and 20 mg ephedrine). A “typical” parturient was defined as having a BMI of 30 kg/m^2^, receiving spinal anesthesia 7 min before treatment of SAH and a single dose of C/T or ephedrine. Incision and uterotomy occurred 5 and 9 min after treatment of SAH, respectively, and spinal block height was thoracic segment 5. Pre-surgery levels of HR, MAP, and SBP were 92 bpm, 100 mmHg, and 139 mmHg, respectively. Baseline levels of HR, MAP, SBP, and DBP were 84 bpm, 64 mmHg, 92 mmHg, and 50 mmHg, respectively.

**Figure 4:**
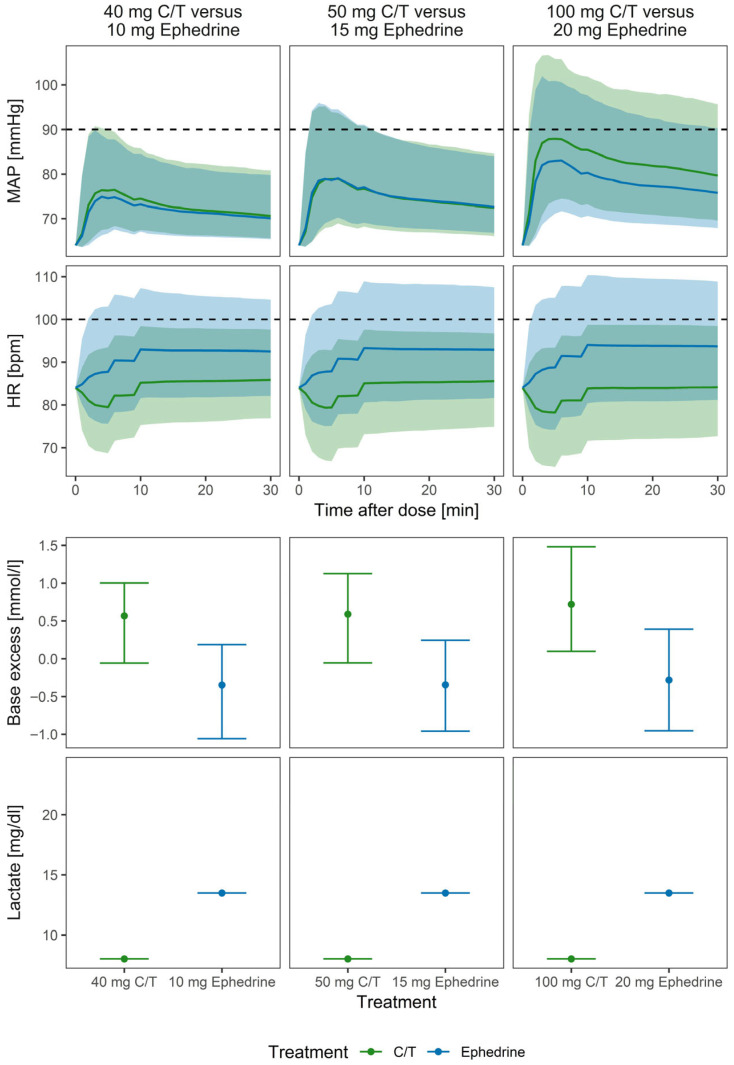
Simulated profiles of MAP, HR and BE for the most commonly applied doses for C/T (green) and ephedrine (blue) in the typical patient. Full lines and points indicate simulated median. Bands and whiskers indicate the 10th and 90th percentiles. Dotted black lines indicate the threshold for hypotension (90 mmHg) and tachycardia (100 bpm).

Simulations indicated similar MAP responses for the most commonly used doses (50 mg C/T and 15 mg ephedrine) (Figure 4). Due to the lower EC50, the HR did not exhibit a strong dose–response across the simulated dose range. However, the HR increase was more pronounced after 15 mg ephedrine, resulting in tachycardia (HR > 100 bpm) in 30.4% of the parturients compared with 6.5% after 50 mg C/T (*p* < 0.001). Furthermore, BE was significantly lower (*p* < 0.001), and lactate was substantially higher after ephedrine treatment.

## 4. Discussion

In this study, we developed a K/PD model describing the hemodynamic effects of C/T and ephedrine on MAP, SBP, and HR in parturients undergoing elective cesarean section. In parallel, we assessed associations between maternal hemodynamics and neonatal outcomes, comprising UA pH, BE and lactate levels, while accounting for maternal, neonatal, surgical and anesthesia-related factors.

The non-interventional study design was chosen to ensure that intra-operative hypotension was managed according to routine practice and the anesthesiologist’s clinical judgment, taking the individual patient’s condition into account [6,8]. Consequently, the study does not influence treatment decisions regarding timing, dose selection, repeat boluses, or additional hemodynamic interventions. While this limits the direct comparability of individual results, it ensures that the emergency situation of hypotension is treated without the disadvantage of the predetermined dosing regimens common within blinded clinical trials. Within this setting, non-linear mixed-effects (NLME) modeling offers a robust framework to characterize time-varying hemodynamic responses under variable dosing patterns and IIV.

The primary analysis of the HYPOTENS results used univariate approaches and reported a higher incidence of tachycardia and a larger HR increase with ephedrine (*p* < 0.01), alongside a reduced need for additional IV boluses (*p* < 0.01) with C/T [4]. While this analysis also highlighted better neonatal outcomes in terms of base deficit and lactate after C/T (*p* < 0.01) [4], the underlying physiological mechanisms remained unclear due to the limitations of univariate analyses. In contrast, our NLME model incorporated full hemodynamic time profiles across the 30 min observational period and accommodated repeated dosing, which occurred in 64.3% of parturients, typically administered two minutes after the initial bolus. Covariate-informed simulations further enabled a more direct comparison of C/T and ephedrine by adjusting for relevant patient- and procedure-related factors.

Our modeling results suggest that 50 mg C/T and 15 mg ephedrine have comparable effects on blood pressure, with a rapid onset and sustained response. However, ephedrine was associated with a 15% greater increase in HR (*p* < 0.001), leading to a higher incidence of tachycardia if the impact of confounding covariates is accounted for (30.4% vs. 6.5% for C/T, *p* < 0.001). Maternal hemodynamic responses were influenced by pre-anesthesia and baseline blood pressure and HR, BMI, spinal block height, and surgery-related stimuli such as incision and uterotomy. Notably, pre-anesthesia and baseline SBP and MAP were key predictors of response, emphasizing the importance of individualized dosing that considers the patient’s hemodynamic profile at the time of hypotension.

Model parameters were estimated with good precision, and GOF diagnostics and visual predictive checks indicated that the model adequately captured the central tendency and variability of the observed data at the population level. However, residual variability was relatively high for HR, with a proportional residual error of 13%CV. Accordingly, predictions and simulations reproduced overall HR trajectories but were less able to capture abrupt, short-lived fluctuations, such as distinct spikes or drops over time. This likely reflects the high sensitivity of HR to transient intraoperative events. Consistent with this, incision and uterotomy were associated with a larger relative increase in HR (3.2%) compared with SBP (2.0%).

In our multiple regression analysis, treatment with ephedrine and elevated maternal HR response following administration of C/T and ephedrine were associated with decreased BE. Adverse neonatal outcomes, including reduced UA pH, are often attributed to the placental transfer of vasoactive agents and fetal metabolic effects [15]. Ephedrine, for example, has been shown to cross the placenta more rapidly than phenylephrine and is associated with increased fetal lactate, glucose, and catecholamines [15]. This suggests the hypothesis that neonatal acid-base depression following ephedrine may be mediated by β-adrenergic stimulation and associated metabolic changes [15]. In this context, maternal tachycardia can be regarded as an indirect marker of exposure to a more pronounced adrenergic state, underscoring the importance of correcting maternal hypotension while avoiding excessive HR elevation. Regarding the impact of treatment with ephedrine, our findings align with the primary analysis results of the HYPOTENS study [4]. However, by using a multifactorial approach, we were able to identify the specific connection of MAX_HR_ and neonatal outcomes, thereby reinforcing the advantages of C/T.

Beyond maternal treatment type and MAX_HR_, several additional predictors of neonatal outcomes were identified. The duration of MAP below pre-surgery levels and spinal block height were significant predictors of pH and BE. A shorter duration of MAP reduction was associated with higher pH and BE, supporting the notion that prolonged maternal hypotension contributes to neonatal umbilical acidosis [2]. Similarly, higher spinal block levels correlated with lower pH and BE, plausibly due to greater sympathetic blockage and a higher likelihood of hypotension with reduced uteroplacental perfusion. While previous studies have reported similar trends, our results provide statistically significant evidence supporting these associations [16,17].

We also observed that the newborns of parturients who received higher doses of the local anesthetic bupivacaine had significantly lower BE and higher lactate levels. Specifically, a 1 mg increase in bupivacaine dose was associated with a BE decrease of 0.186 mmol/L and a lactate increase of 1.05 mg/dL. For BE, this effect was identified in addition to the impact of the duration of hypotension. Although bupivacaine dose did not show an identifiable effect on maternal hemodynamics in the K/PD model, the neonatal association is biologically plausible, given the well-recognized relationship between higher local anesthetic doses, higher sympathetic blockade, and increased risk of hypotension [18].

Pregnancy duration was another significant factor affecting neonatal outcomes, with pH decreasing at a rate of −0.000822 per day, i.e., predictions ranging from 7.36 after 198 pregnancy days to 7.29 after 287 days in the study cohort. This finding is consistent with previous studies that identified a similar linear correlation with a slope of −0.00096 per day, resulting in a pH difference of 0.085 for the same 89-day range [3]. The physiological basis remains uncertain, but proposed explanations include the gradual shift from fetal hemoglobin (HbF) to adult hemoglobin (HbA) late in pregnancy and changes in fetal carbon dioxide handling [19,20]. In contrast to some prior reports suggesting that adjustment of all acid–base biomarkers for gestational age, we did not identify consistent gestational-age effects on BE or lactate [3,20].

Additionally, BE was positively correlated with maternal weight, indicating that higher weight may be beneficial for neonatal acid-base balance. This result contrasts with earlier studies that identified higher BMI as a risk factor for maternal hypotension and fetal acidosis [21,22]. Differences in populations, clinical management, and modeling strategies may contribute to this discrepancy, which warrants further investigation.

The time interval between cord clamping and blood sampling was strongly associated with lactate levels, with a slope of 0.708 mg/dL per minute, resulting in an increase from 12.9 mg/dL to 39.1 mg/dL for the delay range of 0–37 min observed during the study. This finding aligns with prior research that reported a similar increase of 0.559 mg/dL per minute with delayed sampling, which would indicate an increase of 20.7 mg/dL during 37 min [23]. Additionally, lactate levels have been shown to increase when blood samples are stored at room temperature, which may partly explain the variability in our measurements [3]. Standardized reporting and handling procedures for blood gas analyses would likely improve comparability across studies.

Finally, a lower estimated elimination rate parameter in the K/PD model was associated with higher neonatal BE, suggesting that prolonged maternal hemodynamic response may be beneficial, in addition to the duration of hypotension. However, within the K/PD modeling framework, the elimination parameter represents a functional descriptor of the time course of drug effect rather than true systemic clearance. As a result, this association should not be interpreted causally and may reflect unmeasured factors influencing response duration.

Several limitations should be considered when interpreting these results. First, the non-randomized, observational design and reliance on surrogate biochemical endpoints limit causal interpretation and leave the possibility of residual confounding. As such, the findings should be viewed as hypothesis-generating rather than confirmatory.

Additionally, the incidence of fetal acidosis in this study was low: only 12 newborns (5.3%) had a pH < 7.2. This included four (3.2%) and eight (7.7%) newborns treated with C/T and ephedrine, respectively, although the difference was not statistically significant (*p* = 0.133). No neonates had BE or lactate values exceeding the critical thresholds of −12 mmol/L and 90 mg/dL, respectively. Thus, although differences between treatments were identified, both C/T and ephedrine appeared effective in managing SAH sufficiently to avoid severe neonatal biochemical derangements in this cohort.

Furthermore, lactate, BE, and pH are surrogate biochemical markers rather than direct clinical outcomes. While they are well-established indicators of neonatal metabolic condition and peripartum stress and are routinely used in obstetric research as early markers of impaired oxygenation [2,3], the absence of longer-term neonatal follow-up precludes conclusions regarding sustained clinical impact. Future studies should aim to link biochemical differences to clinically meaningful neonatal and neurodevelopmental outcomes.

Despite the robust model development and validation, the description of the maternal hemodynamics has several limitations. The lack of PK data necessitated a simplified K/PD model. More complex kinetic structures (e.g., multi-compartment models or non-linear elimination) cannot be reliably supported without PK data and would introduce parameters that are not identifiable from PD data alone. As a consequence, kinetic parameters should be interpreted as empirical descriptors of the time scale of drug action, rather than true PK quantities. Their role is to govern the delay and persistence of the PD effect, not to quantify systemic drug exposure. Concurrently, substantial IIV remained unexplained by the evaluated covariates. This may partly reflect the absence of PK data, which limits the ability to distinguish variability in drug disposition from variability in pharmacodynamic sensitivity. Within the K/PD framework, the kinetic component represents a hypothetical exposure rather than measured concentrations, and covariate effects that would typically act on PK parameters may therefore be weakly identifiable or absorbed into random effects. Consequently, PK-related sources of variability cannot be explicitly separated, potentially contributing to elevated unexplained IIV and limiting both the detection of mechanistically interpretable covariate relationships as well as the precision of individual-level predictions. Nonetheless, the model remains informative at the population level by capturing the central tendency and overall variability of the pharmacodynamic response. Therefore, it is still useful for comparative purposes, hypothesis generation, and study design, especially when considering that equivalent doses of C/T and ephedrine have not yet been described [7,24].

Furthermore, lactate measurements were available for only a subset of newborns, limiting generalisability for this endpoint. Finally, while we identified several significant predictors of neonatal acidosis, the overall variance explained by these models was modest, particularly for pH. This suggests that other unmeasured factors may also play a critical role and should be investigated in future research. While another limitation might have arisen from the observation period for maternal blood pressure being limited to 30 min, 98.4% of newborns (239 of 243) were born within this time frame, with only one newborn after more than 33 min.

## 5. Conclusions

While C/T and ephedrine both effectively manage maternal hypotension, parturients treated with ephedrine had higher rates of tachycardia. Concurrently, lower maximum maternal HR, shorter duration of maternal hypotension, and treatment with C/T were associated with beneficial changes in BE, lactate, and/or pH in the newborns. In conclusion, our analyses confirmed the importance of effective maternal antihypotensive management, while indicating that newborns potentially benefit from substances that have a lower impact on maternal HR.

## Figures and Tables

**Table 1:** Summary of demographic characteristics of parturients and newborns stratified by treatment arm.

Parameter		C/T (n = 135)	Ephedrine (n = 108)	*p*-Value
Parturient				
Age (years)		33.7 ± 5.35	33.1 ± 5.07	0.39 *
Weight (kg)		87.1 ± 19.2	88.2 ± 20.4	0.69 *
Height (cm)		166 ± 6.89	166 ± 6.95	0.49 *
BMI (kg/m^2^)		31.3 ± 6.13	32 ± 7.11	0.43 *
ASA physical status	I II III	45 (33.3%)	36 (33.3%)	
		89 (65.9%)	71 (65.7%)	0.99 **
		1 (0.74%)	1 (0.93%)	
Newborn				
Gestation (days)		263 ± 9.74	261 ± 10.6	0.27 *
Weight (g)		3440 ± 538	3290 ± 550	0.03 *
Sex	Female Male	57 (42.2%)	51 (47.2%)	0.52 **
		78 (57.8%)	57 (52.8%)	

Data presented as mean ± SD or n (%). * obtained from *t*-test, ** obtained from χ^2^ testing; C/T = cafedrine/theodrenaline; BMI = body mass index; ASA = American Society of Anesthesiologists.

**Table 2:** Summary of hemodynamic, surgery-related and newborn outcome variables.

Parameter		C/T (n = 135)	Ephedrine (n = 108)	*p*-Value
Pre-surgery maternal hemodynamics				
Mean arterial pressure (mmHg)		101 ± 13.9	101 ± 14.2	0.72 *
Systolic blood pressure (mmHg)		139 ± 18.6	138 ± 18.3	0.44 *
Heart rate (bpm)		92.6 ± 14.9	91.7 ± 15.3	0.67 *
Baseline maternal hemodynamics (at the time of SAH diagnosis)				
Mean arterial pressure (mmHg)		64.2 ± 13.5	64.5 ± 9.87	0.81 *
Systolic blood pressure (mmHg)		92.3 ± 15.6	93 ± 12.8	0.72 *
Heart rate (bpm)		85.4 ± 22.1	87.5 ± 22.1	0.47 *
Duration of MAP < pre-surgery MAP (min) ^+^		10.1 ± 6.16	10.2 ± 5.76	0.94 *
Newborn outcome parameter ^++^				
Umbilical cord arterial pH		7.32 ± 0.051	7.31 ± 0.070	0.36 *
Umbilical cord arterial base excess (mmol/L)		−0.933 ± 2.1	−1.94 ± 2.38	0.002 *
Lactate (mg/dL)		18.3 ± 6.83	24.7 ± 13.9	0.085 *
Dose (C/T doses are expressed as cafedrine equivalents)				
Median (range) (mg)		50 (10–200)	15 (5–40)	n.a.
Cumulative dose (mg)		125 ± 76.5	32.8 ± 22.7	n.a.
Number of administrations	1 2 ≥3	51 (37.8%)	38 (35.2%)	0.046 **
		49 (36.3%)	27 (25%)	
		35 (25.9%)	43 (39.8%)	
Surgery-related parameters				
Bupivacaine amount (mg)		10.5 ± 2.44	10.2 ± 01.95	0.19 *
Spinal block height (Thoracic segment)		5.79 ± 1.93	5.02 ± 1.22	<0.001 *
Time cord clamping to blood sampling (min)		7.81 ± 6.64	4.65 ± 5.44	<0.001 *

Data presented as mean ± SD or n (%) unless stated otherwise. * obtained from *t*-test, ** obtained from χ^2^ testing, ^+^ until cord clamping, ^++^ excluding newborns born before cord clamping. C/T = cafedrine/theodrenaline; MAP = mean arterial pressure, n.a = not applicable.

## Data Availability

Qualified researchers may request access to patient-level data and related study documents, including the study protocol and the statistical analysis plan. Requests will be reviewed for scientific merit, product approval status, and conflicts of interest. Patient-level data will be de-identified and study documents will be redacted to protect the privacy of trial participants and to protect commercially confidential information. Please email USMedInfo@tevapharm.com to make your request.

## Abbreviations

The following abbreviations are used in this manuscript:

ASA	American Society of Anesthesiologists
AUC	Area under the curve
BE	Base excess
BMI	Body mass index
BL	Baseline
CL	Clearance
C/T	Cafedrine/Theodrenaline
DBP	Diastolic blood pressure
EBE	Empirical Bayes estimates
EC50	Half-maximum effective concentration
GOF	Goodness-of-fit
HR	Heart rate
IIV	Inter-individual variability
IV	Intravenous
K/PD	Kinetic/pharmacodynamic
MAP	Mean arterial pressure
NLME	Non-linear mixed-effects
PD	Pharmacodynamics
PK	Pharmacokinetics
PRE	Pre-surgery
RSE	Relative standard error
SAH	Spinal anesthesia-induced hypotension
SBP	Systolic blood pressure
UA	Umbilical arterial
V	Volume of distribution

## Appendix A

### Appendix A.1. Maternal Hemodynamic Model

The maternal hemodynamic parameters, including SBP, MAP, and HR, following C/T and ephedrine administration, were described by the following equations.

d⁄dt(A1) = −kel × A1 (A1)

d⁄dt(A2) = −ktr1 × (A1/V − A2) (A2)

d⁄dt(A3) = −ktr1 × (A2 − A3) (A3)

d⁄dt(A4) = −ktr1 × (A3 − A4) (A4)

d⁄dt(A5) = −ktr2 × (A1/V − A5) (A5)

d⁄dt(A6) = −ktr2 × (A5 − A6) (A6)

d⁄dt(A7) = −ktr2 × (A6 − A7) (A7)

d⁄dt(A8) = −ktr2 × (A7 − A8) (A8)

d⁄dt(A9) = −kdel × A9 (A9)

CONC = A4 + A9 (A10)

A = e^(−kdel × TIME_AN_) − A9 (A11)

HR = BL_HR_ + Emax_HR_ × CONC/(CONC + EC50_HR_) + Eff_HR_ × (I + U) (A12)

MAP = BL_MAP_ + Emax_MAP_ × CONC/(CONC + EC50_MAP_) + Eff_BP_ × A + Eff_PP_/3 × (I + U) (A13)

SBP = BL_SBP_ + Emax_SBP_ × CONC/(CONC + EC50_SBP_) + Eff_BP_ × A + Eff_PP_ × (I + U) (A14)

Here, dosing of C/T or ephedrine occurs in compartment A1, hence Equation (A1) depicts the one-compartment kinetic model after IV bolus application. The elimination rate kel is derived by dividing the clearance (CL) by the volume of distribution (V). The kinetic model is followed by three and four transit compartments for the long-term and short-term delayed effects (Equations (A2)–(A4) and (A5)–(A8), respectively). Equations (A9) and (A11) depict the derivation of the time-dependent anesthesia effect, where the hypothetical amount of 1 is dosed in compartment A9 at the time of anesthesia. For the total anesthesia effect at time t, the amount in A9 is subtracted from the baseline effect in dependence of the time between anesthesia and antihypotensive treatment (TIME_AN_). Finally, Equations (A12)–(A14) depict the Emax effect models for each hemodynamic parameter. Here, BL_i_ represents the parameter levels at the time of antihypotensive treatment. Emax_i_ is the difference between MAX_i_ and BL_i_. I and U are covariates with the levels 0 before and 1 after incision and uterotomy occur, respectively. All other parameter meanings are detailed in Appendix A.1 Table A1. All parameters were estimated precisely, with RSEs < 50% and with low shrinkage (<30%).

**Table A1:** Hemodynamic model parameter estimates.

Description	Parameter [Unit]	Estimate (RSE)	IIV [%CV] (RSE)
Fixed effects and IIV			
Clearance	Cl [L/min]	0.0971 (11.2%)	66.9 (9.15%)
Volume of distribution	V [L]	1 (fixed)	77.5 (13%)
Maximum HR	MAX_HR_ [bpm]	77.8 (2.1%)	15.8 (7.35%)
Half-effective concentration HR	EC50_HR_ [mg/L]	5.63 (46.9%)	
Fractional EC50 change for Ephed.	F_EC50 [-]	−0.711 (4%)	
Maximum MAP	MAX_MAP_ [mmHg]	119 (4.8%)	16.5 (15.4%)
Half-effective concentration MAP	EC50_MAP_ [mg/L]	61.7 (23.3%)	
Maximum SBP	MAX_SBP_ [mmHg]	169 (4.7%)	13.2 (18.2%)
Half-effective concentration SBP	EC50_SBP_ [mg/L]	64.4 (23.4%)	
Transit rate long-term effect	Ktr1 [1/min]	0.107 (7.3%)	
Transit rate short-term effect	Ktr2 [1/min]	1.76 (5.9%)	69.1 (5.77%)
Covariate Effects			*p*-value
Fractional MAXHR change for E	F_MAXHR [-]	0.15 (16.6%)	4.0 × 10^−10^
Pre-surgery HR on MAXHR	MAXHR (HR) [-]	0.399 (16.9%)	5.9 × 10^−9^
Pre-surgery MAP on MAXMAP	MAXMAP (MAP) [-]	0.499 (15.5%)	1.9 × 10^−11^
Baseline MAP on MAXMAP	MAXMAP (MAPBL) [-]	−0.53 (23.8%)	5.7 × 10^−17^
Baseline DBP on V	V (DBPBL) [-]	−1.49 (23.2%)	1.6 × 10^−6^
BMI on clearance	Cl (BMI) [-]	−0.948 (32%)	9.4 × 10^−5^
Pre-surgery SBP on MAXSBP	MAXSBP (SBP) [-]	0.577 (13.5%)	9.6 × 10^−16^
Baseline SBP on MAXSBP	MAXSBP (SBPBL) [-]	−0.494 (26.9%)	1.8 × 10^−14^
Rate of BP effect of anesthesia	Kdel [1/min]	0.171 (47.3%)	9 × 10^−19^
Anesthesia effect on BP	EffBP [mmHg]	−9.78 (29.7%)	9 × 10^−19^
Incision/uterotomy effect on HR	EffHR [bpm]	2.75 (18.9%)	3.4 × 10^−23^
Incision/uterotomy effect on PP	EffPP [mmHg]	2.39 (21.7%)	8.6 × 10^−14^
Spinal block height effect on EffBP	EffBP (SPINAL) [-]	−2.05 (44.4%)	3.8 × 10^−6^
Residual variability			
Proportional error HR	PEHR [%CV]	13 (5.4%)	
Proportional error MAP	PEMAP [%CV]	9.37 (6.1%)	
Proportional error SBP	PESBP [%CV]	8.55 (6.8%)	

BP, blood pressure; E, Ephedrine; HR, heart rate; IIV, inter-individual variability; MAP, mean arterial pressure; PP, pulse pressure; RSE, relative standard error; SBP, systolic blood pressure.

### Appendix A.2. Newborn Outcome Models

The stepwise regression analysis identified that newborns’ pH levels are significantly influenced by the duration of MAP below presurgery MAP (until cord clamping), pregnancy duration and spinal block height. The model was statistically significant, with F(3;224) = 5.88, *p* = 0.000703, and accounted for 6.06% of the variance in pH, with an adjusted R^2^ of 0.0606. Detailed parameter estimates, including RSEs, *t*-values, and two-tailed *p*-values, are available in Appendix A.2 Table A2. Observations compared with model predictions stratified by predictor are presented in Appendix A.2 Figure A3.

**Table A2:** pH model parameter estimates.

Description	Estimate (RSE)	*t*-Value	Pr(>|t|)
Intercept [-]	7.52 (1.31%)	76.6	<0.001
Duration MAP < pre-surgery MAP [1/min]	−0.00177 (36.2%)	−2.77	0.00613
Pregnancy duration [1/day]	−0.000822 (45.7%)	−2.19	0.0298
Spinal block height [1/Spinal segment]	0.00534 (46.1%)	2.17	0.0309

Pr(>|t|): two-tailed *p*-value for the *t*-value; MAP, mean arterial pressure, RSE: relative standard error.

The stepwise regression analysis for neonatal BE indicated significant effects of the maternal antihypotensive treatment (C/T or ephedrine), spinal block height, bupivacaine dose administered to the parturient, maternal body weight, as well as model parameters for the maximum HR (MAXHR) and elimination rate of C/T or ephedrine (kel). The model was statistically significant with F(8;196) = 8.23, *p* = 1.36 × 10^−9^ and explained 22.1% of the variance in BE, with an adjusted R^2^ of 0.221. Detailed parameter estimates, including RSEs, *t*-values, and two-tailed *p*-values, are available in Appendix A.2 Table A3. Observations compared with model predictions stratified by predictor are presented in Appendix A.2 Figure A4.

**Table A3:** Base excess model parameter estimates.

Description	Estimate (RSE)	*t*-Value	Pr(>|t|)
Intercept [mmol/L]	−2.09 (75.6%)	−1.32	0.188
Treatment with C/T [mmol/L]	3.26 (23.8%)	4.2	<0.001
Duration MAP < pre-surgery MAP [mmol/L/min]	−0.105 (24.6%)	−4.09	<0.001
Treatment with E [mmol/L]	2.64 (29.7%)	3.37	<0.001
Spinal block height [mmol/L/thoracic segment]	0.3 (30.7%)	3.25	0.00134
Bupivacaine amount [mmol/L/mg]	−0.186 (35.5%)	−2.82	0.00536
Weight (mother) [mmol/L/kg]	0.0176 (41.6%)	2.41	0.0171
Model parameter MAXHR [mmol/L/bpm]	−0.0256 (42.6%)	−2.34	0.0202
Model parameter kel [mmol/L×min]	−1.74 (47.6%)	−2.1	0.0368

Pr(>|t|): two-tailed *p*-value for the *t*-value; C/T, cafedrine/theodrenaline, MAP, mean arterial pressure, RSE: relative standard error.

The stepwise regression analysis revealed that neonatal lactate levels were influenced by the time delay between cord clamping and blood sampling, treatment of the parturient with ephedrine (compared with treatment with C/T or none) and the bupivacaine dose administered to the parturient. The model was statistically significant, with F(3;72) = 15.5, *p* = 6.99 × 10^−8^, and explained 36.7% of the variance in lactate, with an adjusted R2 of 0.367. Detailed parameter estimates, including RSEs, *t*-values, and two-tailed *p*-values, are available in Appendix A.2 Table A4. Observations compared with model predictions stratified by predictor are presented in Appendix A.2 Figure A5.

**Table A4:** Lactate model parameter estimates.

Description	Estimate (RSE)	*t*-Value	Pr (>|t|)
Intercept [mg/dL]	3.43 (115%)	0.867	0.389
Cord clamping to blood sampling [mg/dL/min]	0.708 (18.9%)	5.3	<0.001
Treatment with ephedrine [mg/dL]	5.46 (37.2%)	2.69	0.00883
Bupivacaine amount [mg/dL/mg]	1.05 (37.5%)	2.66	0.00967

Pr(>|t|): two-tailed *p*-value for the *t*-value; RSE: relative standard error.
